# Sulforaphane Wrapped in Self-Assembled Nanomicelle Enhances the Effect of Sonodynamic Therapy on Glioma

**DOI:** 10.3390/pharmaceutics17010034

**Published:** 2024-12-30

**Authors:** Yihong Li, Xuejie Yang, Zhen Wei, Heng Niu, Liyang Wu, Caijing Chen, Huina Liu, Ting Cai, Huadong Fan

**Affiliations:** 1 Ningbo No. 2 Hospital, Ningbo 315099, China; weizhen_2015@163.com (Z.W.); niuhengnzh_0411@163.com (H.N.); 13858262522@163.com (C.C.); liuhuina@ucas.ac.cn (H.L.); caiting@ucas.ac.cn (T.C.); 2 Innovation Center for Diagnosis and Treatment of Neurological Diseases, Ningbo Institute of Life and Health Industry, University of Chinese Academy of Sciences, Ningbo 315000, China; yang2804001425@163.com (X.Y.); wly960816@163.com (L.W.); 3 Lab of Nanopharmacology Research for Neurodegeneration, Department of Research and Development of Science and Technology, Ningbo Institute of Life and Health Industry, University of Chinese Academy of Sciences, Ningbo 315000, China; 4 Lab of Dementia and Neurorehabilitation Research, Department of Research and Development of Science and Technology, Ningbo Institute of Life and Health Industry, University of Chinese Academy of Sciences, Ningbo 315000, China

**Keywords:** glioma, sonodynamic therapy, self-assembled nanomicelle, sulforaphane, neutrophil infiltration

## Abstract

**Background/Objectives:** The two obstacles for treating glioma are the skull and the blood brain–barrier (BBB), the first of which forms a physical shield that increases the difficulties of traditional surgery or radiotherapy, while the latter prevents antitumor drugs reaching tumor sites. To conquer these issues, we take advantage of the high penetrating ability of sonodynamic therapy (SDT), combined with a novel nanocomplex that can easily pass the BBB. **Methods:** Through ultrasonic polymerization, the amphiphilic peptides (C_18_GR_7_RGDS) were self-assembled as a spherical shell encapsulating a sonosensitizer Rose Bengal (RB) and a plant-derived compound, sulforaphane (SFN), to form the nanocomplex SFN@RB@SPM. **Results/Conclusions:** SFN@RB@SPM can be internalized by the glioma cells through the tumor-targeting motif RGDS (abbreviated for the peptide sequence composed of arginine, glycine, aspartic acid, and serine), and further executes antitumor function during SDT. Also, SFN@RB@SPM could be easily taken up by U87-MG cells and cross the BBB in glioma-bearing mice during SDT. The mechanism investigation revealed that, compared with the SFN-free nanocomplex (RB@SPM), SFN@RB@SPM induced much more apoptosis of U87-MG cells in an ROS-dependent manner through the depletion of glutathione by SFN and the cavitation effect by SDT. In animal experiments, besides a significant reduction in tumor volume and a delay in losing body weight, H&E staining showed a massive infiltration of neutrophils adjacent to the tumor sites, indicating this novel nanocomplex SFN@RB@SPM can synergistically augment SDT efficacy, partially by enhancing the antitumor function of innate immunity.

## 1. Introduction

Gliomas are the most aggressive brain tumors in adults, with extremely high mortality and recurrence [1,2]. The current routine treatments for this malignancy include a combination of surgery, radiotherapy, and chemotherapy. However, these solutions have a variety of drawbacks or limitations regarding the incomplete surgical ablation of cancerous tissues [3], radio-resistance [4], and low delivery efficiency or weak bioavailability of the chemotherapeutic drugs [5]. Most importantly, the existence of the blood–brain barrier (BBB) prevents most of the active drugs reaching the tumor sites. Hence, it is still urgent to pursue more effective treatments for this malignancy, especially to facilitate the permeability of the BBB, as well to enhance the specificity of drug delivery. Currently, there are two promising solutions to facilitate drug delivery: one approach is equipping the drugs with BBB-crossing nanoparticles (NPs) such as self-assembled nanomicelles [6], while the other is to utilize BBB disruption-enhanced transports. For instance, with the help of focused ultrasound (FUS) in sonodynamic therapy (SDT), the reversibly transient BBB opening can promote the local drug delivery/release and enhance the tumor-killing effect [7,8,9,10,11].

Due to the thermal and mechanical effects of ultrasound, as well as its non-invasive features and high penetrative ability, SDT and its related combination therapies can achieve precise destruction of cancerous tissue through releasing accumulating sonosensitizer or antitumor drugs at tumor sites, with minimal damage to the adjacent tissues [12]. At present, the most widely accepted tumor-killing mechanism of SDT is that the sonosensitizers enhance the ultrasound-induced cavitation effect through facilitating the generation of cellular ROS [13]. Traditional sonosensitizers have poor biocompatibility and off-target effects due to inadequate accumulation inside the tumor, resulting in poor therapeutic efficacy. Most organic sonosensitizers are derived from photosensitizers applied in photodynamic therapy (PDT) [13,14]. However, PDT is unsuitable for treating the tumors of internal organs because of weak penetration of the light to deep tissues [15,16]. Rose Bengal (RB), a well-known sensitizer that has been applied in PDT, exerts a better antitumor effect due to enhanced sonosensitizer activity in SDT [17]. Meanwhile, RB has been reported as an immune-potentiator that activates antitumor responses, as well as generating excessive ROS to induce tumor cell death [18,19]. Therefore, RB has been employed in clinical trials for numerous cancers [20,21,22].

C_18_GR_7_ is an amphiphilic self-assembled peptide that can form spherical nanomicelles in the water phase, which can encapsulate functional payloads like sonosensitizers and antitumor drugs [23]. However, the increased interstitial fluid and the dense stroma of tumor may hinder the passive diffusion and transportation of the nanocomplex [24]. RGDS is a tumor-homing short peptide that can be recognized by α_v_β_3_ integrin expressed at the glioma surface [25,26,27,28]. Thus, to improve the efficiency of targeted delivery and assure the site-specific enrichment of sonosensitizers and antitumor drugs, an RGDS-tagged amphiphilic peptide (C_18_GR_7_RGDS) has been designed [23,29,30,31].

Cancer cells can take advantage of hypoxia and excessive glutathione (GSH) in the tumor microenvironment (TME) to combat SDT-induced ROS for survival, which significantly weakens the SDT efficacy [32]. Therefore, in addition to strategies for enhancing drug delivery and targeting, a combination with antitumor drugs to elevate ROS levels in the TME is indispensable to enhance the SDT effect [13]. Sulforaphane (SFN), a compound derived from plants of the Cruciferae family (e.g., broccoli), has been found to exert an anticancer effect, especially in some primary tumors including CNS tumors [33,34,35,36,37,38]. One of the important tumor-killing mechanisms of SFN is to elevate ROS levels in tumor tissues to induce apoptosis [39]. Furthermore, SFN shows nearly the opposite effect in normal and tumor cells; namely, it is beneficial for normal cells while harmful for tumor cells. Although SFN has been reported to enhance the tumor-killing effect of PDT [40], whether SFN exerts a synergistic effect with SDT remains unclear.

In this study, we developed a novel nanocomplex SFN@RB@SPM, composed of a self-assembled nanomicelle (C_18_GR_7_RGDS) encapsulating RB and SFN, to synergistically enhance the efficacy of SDT. Both in vitro and in vivo experiments indicated that this novel SFN-containing nanocomplex (SFN@RB@SPM) exhibits more robust antitumor activity than the SFN-free one (RB@SPM) during SDT.

## 2. Materials and Methods

### 2.1. Reagents and Kits

The peptide amphiphile (C_18_GR_7_RGDS) was obtained from ChinaPeptides Co., Ltd. (Hubei, China). Sulforaphane (SFN) was bought from LST Laboratories (St. Paul, MN, USA). Rose Bengal (RB) was purchased from Aladdin Co., Ltd. (Shanghai, China). Cell Counting Kit-8 (CCK-8) and H&E staining kit were bought from Beyotime Biotechnology Co., Ltd. (Shanghai, China). TUNEL Assay Apoptosis Detection Kit was purchased from Absin Biotech Co., Ltd. (Shanghai, China). Calcein-AM/PI Live-Dead Cell Staining Kit was bought from Solarbio Life Sciences Co., Ltd. (Shanghai, China). DCFH-DA was purchased from KeyGEN Biotech (Jiangsu, China).

### 2.2. Synthesis of SFN@RB@SPM

The nanomicelle was self-assembled through ultrasound polymerization. Briefly, the solutions of SPM (6 mg/mL), RB (0.6 mg/mL) and SFN (6 mM) were mixed in the volume ratio of 1:1:1, followed by 30 minutes of sonication at 28 kHz and 25 °C. Then, the mixture was dialysed (molecular weight cut off: 1000 Da) for another 48 h to remove free RB and SFN. The encapsulation efficiencies of RB and SFN were determined by using UV–VIS spectrophotometer (Spectra Max, Molecular Devices, San Jose, CA, USA) and high-performance liquid chromatography (HPLC) (Shimadzu LC-20A, Kyoto, Japan), respectively. The nanomicelle (SFN@RB@SPM) was characterized by transmission electron microscopy (Hitachi 7650, Tokyo, Japan). The size, polydispersity index (PDI), and zeta potential of the nanomicelle were measured by DLS (Zetasizer Nano ZS, Malvern Instruments Ltd., Worcestershire, UK).

### 2.3. Cell Culture and Animals

The human U87-MG cells and luciferase-expressing U87 cells were bought from Pricella Life Science & Technology Co., Ltd. (Hubei, China). The cells were cultured in MEM medium containing 10% FBS at 37 °C in a humidified atmosphere containing 5% CO_2_. BALB/c nude mice (6–8 weeks) were obtained from Sibeifu Biotechnology Co., Ltd. (Beijing, China) and housed at room temperature with a 12 h light/dark cycle and allowed free access to food and water. All the animal experiments were approved by the Institutional Animal Care and Use Committee (IACUC) of the Ningbo Institute of Life and Health Industry, UCAS.

### 2.4. Cellular Uptake Experiment

The time-dependent cellular uptake of SFN@RB@SPM in U87-MG cells was recorded by a laser confocal fluorescence microscope (Zeiss LSM900, Oberkochen, Germany). Briefly, U87-MG cells were seeded at a density of 1 × 10^5^ cells/mL in 20 mm dishes. After incubating overnight, the cells were treated with SFN@RB@SPM (at a concentration normalized to 10 μM of SFN or 1 μM of RB) for 30 min, 1 h, and 4 h. Then, the cells were stained with DAPI for 15 min and washed with PBS 3 times before imaging.

### 2.5. In Vitro Cytotoxicity Study

For in vitro SDT treatment, U87-MG cells were randomly divided into eight groups: Control, US, SFN, SFN with US, RB@SPM, RB@SPM with US, SFN@RB@SFN, and SFN@RB@SPM with US. The cells were incubated with the above agents for 4 h, and then subjected to US exposure (1 MHz, 1 W/cm^2^, 30 s). After 24 h, the viability of U87-MG cells with different treatments was determined by CCK-8 assay. Meanwhile, cells subjected to the same treatments as above were fixed with 4% paraformaldehyde solution and stained with Calcein-AM (1.0 μM)/PI (2.0 μM) to directly visualize the proportion of dead/live cells.

### 2.6. Cell Apoptosis Study

The number of apoptotic cells was determined by flow cytometry using a FITC/Annexin V apoptosis detection kit (BD Bioscience, Franklin Lakes, NJ, USA), following the manufacturer’s instructions. Briefly, U87-MG cells were pretreated with Control, SFN, RB@SPM, and SFN@RB@SPM for 4 h, followed by either ultrasound exposure for 30 s (1 MHz, 1 W/cm^2^, 50% duty cycle), or no exposure. After 24 h, the control and treated cells were collected, washed twice with PBS, resuspended in binding buffer and then incubated with Annexin V and PI for 15 min. The apoptotic cells were detected using a FACSCalibur flow cytometer (DxFLEX, Beckman, IN, USA). The raw data were analyzed using FlowJo 7.6 software.

### 2.7. Estimating the Generation of Cellular ROS

Dichlorofluorescein (DCF) was used to assess intracellular ROS levels. Briefly, U87-MG cells were treated according to the same protocol as described above. After 24 h, the cells were incubated with 10 μM of DCHF-DA for 20 min. Finally, the cells were washed with PBS 3 times before being visualized under a laser confocal fluorescence microscopy (Zeiss LSM900, Oberkochen, Germany).

### 2.8. Measurement of Intracellular GSH

U87-MG cells were seeded in petri dishes for 24 h. Then the cells were pretreated with Control, SFN (10 μM), RB@SPM (normalized to the dose of 1 μM RB), SFN@RB@SPM (normalized to the dose of 10 μM SFN or 1 μM RB) for 4 h, followed by either US exposure for 30 s (1 MHz, 1 W/cm^2^, 50% duty cycle) or no exposure. After another 24 h, the cells were collected and the intracellular GSH content was measured using a GSH detection kit (Solarbio, Beijing, China) according to the manufacturer’s instructions. Finally, the GSH level was quantified by the UV–VIS spectrophotometer at a wavelength of 412 nm.

### 2.9. In Vivo Anti-Glioblastoma Experiments

To evaluate the tumor-targeting efficiency of SFN@RB@SPM in vivo, free RB and SFN@RB@SPM were intravenously injected into the luciferase-expressing U87 tumor-bearing BALB/c nude mice. At different time points post injection (0.5 h, 1 h, 2 h, 4 h, 6 h, 8 h), the fluorescence images and bioluminescence images were taken by IVIS Spectrum imaging system (Perkin Elmer, Shelton, CT, USA) to evaluate the tumor-targeting efficiency of SFN@RB@SPM. To evaluate the antitumor efficacy, the luciferase-expressing U87 tumor-bearing BALB/c nude mice were randomly divided into 6 groups (*n* = 6) as follows: PBS, PBS with US, RB (1 mg/kg) with US, SFN (1.77 mg/kg) with US, RB@SPM (normalized to the dose of 1 mg/kg RB) with US, and SFN@RB@SPM (normalized to the dose of 1.77 mg/kg SFN or 1 mg/kg RB) with US. Besides the PBS-treated group, mice in the other 5 groups were exposed to US (1.0 MHz, 1 W/cm^2^, 50% duty cycle) for 3 min after intravenous injection. The frequency for the above treatments was 1 time per 3 days, and the duration was 13 days. The body weight and tumor area were monitored every 2 days. The tumor area was calculated from the geometric mean diameter using the equation: tumor area = πR^2^. The in vivo tumor area was determined using IVIS Spectrum (Perkin Elmer, Shelton, CT, USA). On day 20, the mice were sacrificed and the brain tissues were isolated for histological experiments (TUNEL and H&E staining).

### 2.10. Statistical Analysis

Statistical analyses were performed using GraphPad Prism 5.0 software (GraphPad, San Diego, CA, USA). All data were expressed as means ± SEM of at least 3 independent experiments. All data from this study were analyzed by One-way ANOVA. Multiple comparison post-tests between groups were conducted using Bonferroni’s comparison test. A *p*-value < 0.05 was considered statistically significant.

## 3. Results

### 3.1. Fabrication and Characterization of SFN@RB@SPM

**Figure 1:**
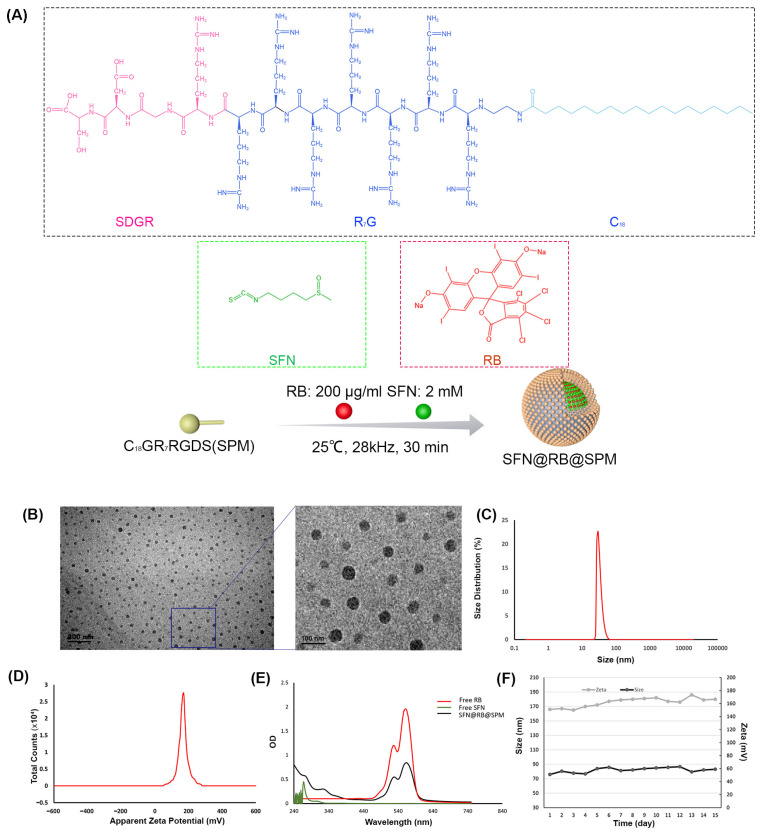
Characterization of self-assembled nanocomplex SFN@RB@SPM. (**A**) Chemical structures of C_18_GR_7_RGDS (SPM), RB, and SFN. For the molecular composition of SPM, as illustrated, the pink hydrophilic sequence SDGR is conjugated to the green hydrophobic sequence C_18_ through the blue linker sequence R_7_G. At the reaction concentration of 200 μg/mL of RB and 2 mM of SFN, after being sonicated (25 °C, 28 kHz for 30 min) for polymerization, RB and SFN could be encapsulated into the self-assembled nanomicelle (SPM) to form a new nanocomplex, SFN@RB@SPM. (**B**) The nanocomplex SFN@RB@SPM was visualized by transmission electron microscope with 80,000× magnification. Scale bar, 200 nm for the left photo and 100 nm for the right one. (**C**) Size distribution indicates the mean size of SFN@RB@SPM is approximately 57 nm. (**D**) The apparent zeta potential of SFN@RB@SPM is 166 ± 0.81 mV. (**E**) UV–VIS absorption experiment manifested the specific peaks for SFN@RB@SPM, confirming that the free SFN and RB had been successfully enveloped into SPM. (**F**) The size and zeta potential were measured every day for 15 days, with the results indicating good water stability.

We have previously synthesized RB@SPM, a self-assembled peptide nanomicelle (SPM) encapsulated with the sonosensitizer RB. To further improve the efficacy of SDT, using similar protocols [28], we simultaneously loaded SFN and RB into SPM. As shown in Figure 1A, SPM is composed of amphiphilic peptides (C_18_GR_7_RGDS) that tend to polymerize due to the hydrophobic interaction at C_18_ terminals. Through ultrasound polymerization, SFN and RB were encapsulated into the hydrophobic core of SPM, forming a core–shell structured nanocomplex SFN@RB@SPM. The hydrophobic core of SFN@RB@SPM can increase the solubility of SFN and RB. As shown in Figure 1B,C, transmission electron microscopy (TEM) and dynamic light scattering (DLS) experiments indicated that SFN@RB@SPM formed a spherical structure with diameter of ~57 nm, which was slightly larger than that of RB@SPM (50 nm, Figure S1). The results also revealed that SFN@RB@SPM exhibited a higher zeta potential (166 ± 0.81 mV in Figure 1C) than that of RB@SPM (132 ± 0.74 mV, Figure S1). The UV–VIS absorption test verified that SFN and RB were successfully loaded into SPM (Figure 1E). The molar ratio of SFN/RB in the nanocomplex of SFN@RB@SPM is 10/1, calculated by acquiring the concentrations of SFN and RB via high-performance liquid chromatology (HPLC) and ultraviolet–visible spectrophotometry (UV-VIS), respectively. After monitoring for 15 days, we found that SFN@RB@SPM displayed excellent stability of size and zeta potential (Figure 1F).

### 3.2. SFN@RB@SPM Can Be Efficiently Internalized into U87-MG Cells via the Tumor-Targeting Motif RGDS

**Figure 2:**
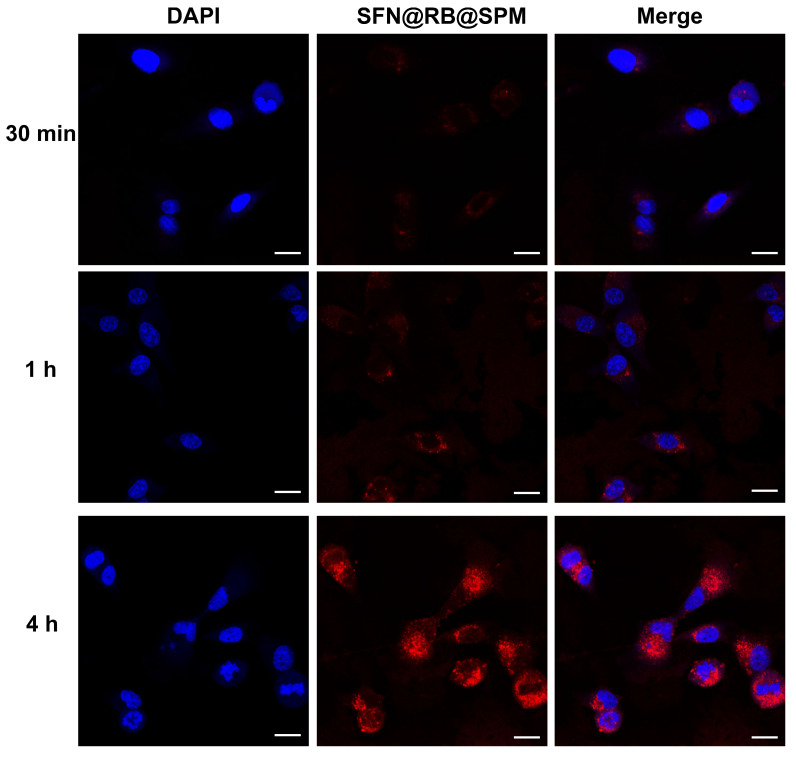
Cellular uptake of SFN@RB@SPM. U87-MG cells were treated with SFN@RB@SPM at the concentration of 10 μM (normalized to the molar concentration of SFN) for 30 min, 1 h, and 4 h, and then the internalization of SFN@RB@SPM (monitored by red fluorescence of RB) by U87-MG cells was visualized under a laser confocal fluorescence microscope. The nuclei were stained with DAPI (blue). Scale bar, 20 μm.

To examine whether SFN@RB@SPM can efficiently enter cells, a cell uptake test was performed. U87-MG cells were treated with 10 μM of SFN@RB@SPM (a molar concentration that was normalized to the payload of SFN). After 30 min, as shown in Figure 2, only a small number of SFN@RB@SPM particles had passed through the cell membrane and a very weak red fluorescence could be visualized. At the timepoint of 1 h, the red fluorescence became more intense. As the incubation time reached 4 h, many aggregations appeared in the cytoplasm, with a peak intensity in the red fluorescence. These results indicate that the internalization of SFN@RB@SPM by U87-MG cells in vitro occurred in a time-dependent manner. Because RGDS-tagged amphiphilic peptide (SPM) was also designed to have a tumor-targeting function, as mentioned previously, we also observed the targeting efficiency of SFN@RB@SPM in the U87 glioma xenograft mice model. We found that SFN@RB@SPM rapidly accumulated around the tumor tissue starting from 30 min and reached almost peak concentration at 2 h after the intravenous injection (Figure S2). Considering that the nanocomplex can efficiently enter U87-MG cells in vitro or in vivo, we speculate that the tag of RGDS in SPM may play a key role in tumor integrin α_v_β_3_ receptor-mediated endocytosis.

### 3.3. SFN@RB@SPM Exhibits a Synergistic Effect with SDT In Vitro

**Figure 3:**
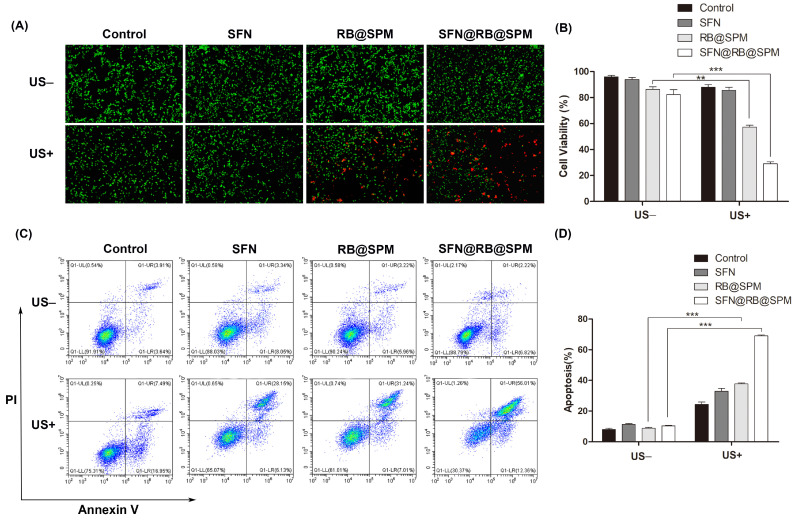
SFN@RB@SPM shows synergistic cytotoxicity with US irradiation in vitro. (**A**) The upper panel shows that U87-MG cells were treated with control, SFN (10 μM), RB@SPM (1 μM), or SFN@RB@SPM (normalized to the molar concentration of 10 μM SFN) with or without US irradiation for 24 h, where US (ultrasound irradiation) refers to high energy ultrasound waves exciting a sonosensitizer, resembling a process of light irradiation in PDT. In contrast, the lower panel shows that U87-MG cells were pretreated with control, SFN (10 μM), RB@SPM (1 μM) or SFN@RB@SPM (normalized to the molar concentration of 10 μM SFN) for 4 h and then exposed to US (1 MHz, 1 W/cm^2^) for 24 h. Then the cells were stained with Calcein-AM to show live (green) and dead (red) cells. Scale bar, 100 μm. (**B**) After the same treatment procedures as in (**A**), an MTT assay was conducted to determine cell viability. The data represent the mean ± SEM of three independent experiments. ** *p <* 0.01, *** *p <* 0.001 versus control. (**C**) U87-MG cells were subjected to flow cytometry to detect the apoptosis induced by SFN, RB@SPM, and SFN@RB@SPM with or without US irradiation (1 MHz, 1 W/cm^2^). The cells were double-stained with Annexin V/PI, and the apoptotic ratio was quantified in (**D**), expressed as mean ± SEM of three independent experiments. *** *p* < 0.001 versus control.

To investigate whether SFN@RB@SPM has an enhanced cytotoxic effect in combination with SDT, we performed Calcein-AM/PI staining to directly observe the ratio of live/dead cells and also conducted a CCK-8 assay to assess the toxicity of SFN, RB@SPM and SFN@RB@SPM in vitro. As mentioned above, SFN and RB have a fixed molar ratio of 10/1 in the nanocomplex of SFN@RB@SPM, so the concentrations we used to treat U87-MG cells were normalized to the molar concentration of 10 μM of SFN or 1 μM of RB. As expected, under the conditions of no US irradiation (the upper panel in Figure 3A), there were seldom dead cells after treatment of control, SFN, RB@SPM or SFN@RB@SPM. However, in combination with US irradiation (the lower panel in Figure 3A), RB@SPM resulted in a small numbers of cell death, whereas SFN@RB@SPM resulted in a large portion of dead cells. A previous study by Liu’s group reported that another nanocomplex containing RB of 40 μM exerted an obvious cytotoxicity [41]. However, our study indicated that even at the low dose of 1 μM, RB in SFN@RB@SPM can achieve the most significantly toxic effect toward U87-MG cells (the right bottom picture in Figure 3A), suggesting that SFN plays a pivotal role in SFN@RB@SPM-induced cytotoxicity during SDT. Although RB@SPM could inhibit cell viability, SFN@RB@SPM significantly enhanced such an effect under US irradiation (Figure 3B). Flow cytometry experiments were also conducted to evaluate the ratio of cell apoptosis. As shown in Figure 3C,D, SFN@RB@SPM induced the largest ratio (~70%) of cell apoptosis. Together, these results demonstrated that SFN can synergistically enhance the RB@SPM-caused cytotoxic effect during SDT.

### 3.4. SFN@RB@SPM Induces Cytotoxicity During SDT, Which Is ROS-Dependent and Is Partially Mediated Through a GSH-Depletion Mechanism in U87-MG Cells

**Figure 4:**
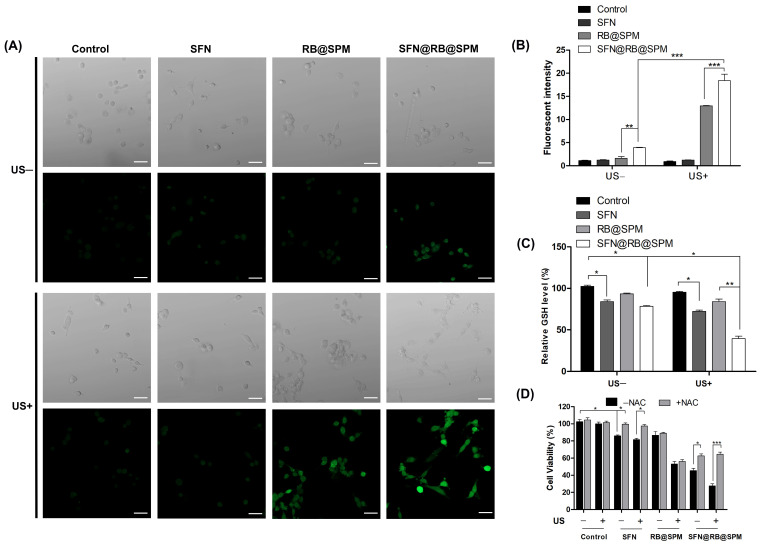
SFN@RB@SPM augments the cytotoxic effect of SDT in vitro in an ROS-dependent manner through the synergism of SFN-mediated GSH depletion and RB-mediated elevation of ROS. (**A**) U87-MG cells were pretreated with control, SFN (10 μM), RB@SPM (1 μM), or SFN@RB@SPM (normalized to the molar concentration of 10 μM SFN) for 4 h, with or without following US irradiation (1 MHz, 1 W/cm^2^) for 30 s. ROS levels were then measured in the cells using Dichlorofluorescein (DCF). As indicated, the intensity of green fluorescence is in proportion with the intracellular level of ROS. Scale bar, 50 μm. (**B**) The quantification of fluorescence intensity in (**A**). Data represent the mean ± SEM of three independent experiments. ** *p* < 0.01, *** *p* < 0.001 versus control. (**C**) U87-MG cells were treated with the same procedures in (**A**), and then the intracellular GSH contents were measured using a GSH detection kit. Data represent the mean ± SEM of three independent experiments. * *p* < 0.05, ** *p* < 0.01 versus control. (**D**) U87-MG cells were pretreated with or without N-acetylcysteine (NAC), and then these two groups were treated in the same manner as in (**A**), and the cell viability was determined 24 h later using CCK8 assay. Data represent the mean ± SEM of three independent experiments. * *p* < 0.05, *** *p* < 0.001 versus control.

It is well accepted that the traditional sonosensitizers usually trigger tumor cell apoptosis through increasing ROS level during SDT. To explore if the novel nanocomplex SFN@RB@SPM also functions by a similar mechanism, we designed an experiment to directly detect the ROS generated in U87-MG cells. As shown in the second panel of Figure 4A, under the conditions of non-US irradiation, compared with control group, the SFN- or RB@SPM-treated groups only generated a trace of ROS, as detected by the ROS probe Dichlorofluorescein, whereas the ROS level in the SFN@RB@SPM-treated group was slightly increased. This might be due to more SFN molecules entering U87-MG cells mediated by the RGDS motif in SFN@RB@SPM. Under SDT, as shown in the fourth panel of Figure 4A, although the RB@SPM-treated group displayed a stronger increase in ROS than that of the control or SFN-treated groups, there is a much higher level of ROS generated in the SFN@RB@SPM-treated group. To further elucidate the mechanism by which SFN facilitates the generation of ROS, we detected the GSH levels in U87-MG cells. As indicated in Figure 4B, either with or without US irradiation, SFN could slightly decrease the GSH level, whereas RB@SPM had no significant influence on the level of GSH. However, SFN@RB@SPM strongly inhibited the GSH level, especially during SDT, suggesting that only SFN in the nanocomplex can counteract GSH to elevate the ROS, whereas RB had no direct effect on cellular GSH contents and may increase ROS through other mechanisms such as a US-induced cavitation effect (e.g., the pyrolysis of H_2_O). Our hypothesis that SFN@RB@SPM-induced ROS-dependent cytotoxicity is partially mediated via GSH depletion in the U87-MG cells is further demonstrated in Figure 4C, which clearly shows that pretreatment with NAC, a precursor of GSH, could strongly rescue SFN or SFN@RB@SPM-induced decline of cell viability, whereas the cell viability of the RB@SPM-treated group had no significant change upon pretreatment with NAC. Taken together, these results suggest that SFN enveloped in SFN@RB@SPM executes its function of cytotoxicity in two ways: one is to increase its cellular concentration by the targeted delivery of SPM, and the other is to elevate the ROS level by depleting GSH, which is mechanistically different from the RB@SPM-induced direct increase in ROS.

Until now, we can conclude that, this new nanocomplex SFN@RB@SPM manifests a promising character with more enhanced cytotoxicity under the assistance of US irradiation compared to the SFN-free nanocomplex (RB@SPM). The potential mechanism is probably that SFN, a key component of the nanocomplex, largely promotes the generation of ROS within the tumor cells and thus enhances ROS-induced cytotoxicity.

### 3.5. SFN@RB@SPM Significantly Inhibits the Growth of Glioma Xenografts in Nude Mice by Enhancing the Antitumor Function of Innate Immunity

**Figure 5:**
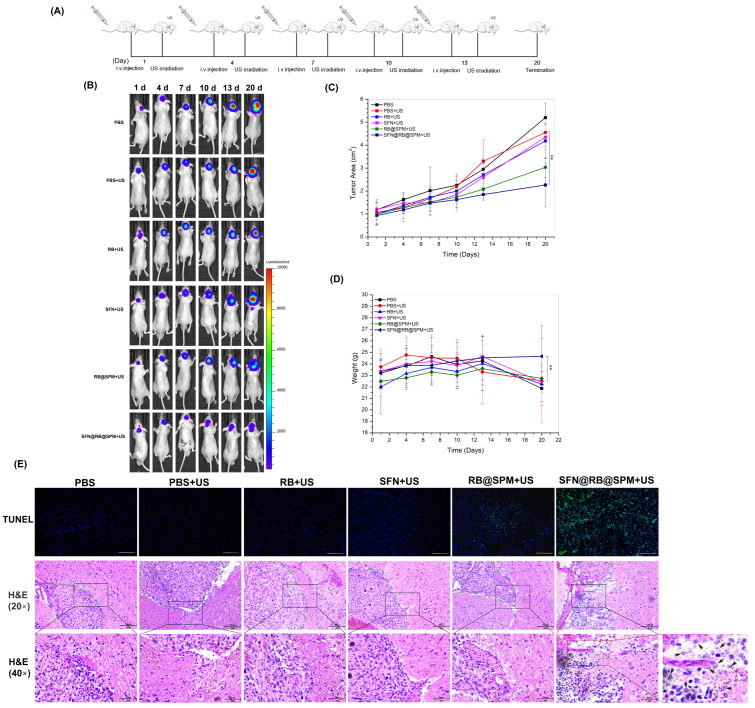
SFN@RB@SPM exhibits a robust antitumor activity for the glioma xenograft of nude mice in coordination with SDT. (**A**) Schematic illustration showing the time window and the route of drug administration in combination with SDT for the glioma-bearing nude mice. Mice were intravenously injected with PBS, RB, SFN, RB@SPM, or SFN@RB@SPM, with or without following US irradiation every 3 days for five repetitions of the treatments. The mice were then sacrificed at day 20. (**B**) Glioma-bearing mice were randomly divided into six groups (*n* = 6) treated with PBS, PBS plus US, RB plus US, SFN plus US, RB@SPM plus US, and SFN@RB@SFN plus US. The bioluminescence was recorded by the IVIS Spectrum imaging system at different time points (1 d, 4 d, 7 d, 10 d, 13 d, 20 d) post treatments. (**C**) The tumor volume in each group of mice was determined using IVIS Spectrum, and the data represent the mean volume of tumor ± SEM in each group of mice. ** *p* < 0.01 versus control. (**D**) The body weight was recorded at the indicated time points, and the data represent the mean ± SEM in each group of mice. ** *p* < 0.01 versus control. (**E**) The mice of the above six groups were sacrificed at day 20, and the brain tissues were isolated for TUNEL (the first panel) and H&E staining (the last two panels). The scale bars of photos from first to third panel are 100 μm, 100 μm and 50 μm, respectively. The green fluorescence represents the apoptotic cells. The middle panel displays images of lower magnification (20×) for H&E staining. The green dotted lines within the images indicate the boundary between the tumor and normal tissues. In the SFN@RB@SPM plus US-treated group of the third panel, a large number of neutrophils have infiltrated (indicated by the arrowheads) into the area adjacent to the tumor tissue. This rectangular area of the 40× image is further magnified.

To further verify whether SFN@RB@SPM exerts an improvement in antitumor effects compared with the traditional formulation of RB@SPM applied in SDT in vivo, we employed the U87-glioma xenograft mice model. As depicted in Figure 5A, besides the control group (PBS without US), the glioma-bearing nude mice in another five groups were exposed to US irradiation after intravenous injection with PBS, RB, SFN, RB@SPM, and SFN@RB@SPM for 2 h, respectively. These treatments were repeated every 3 days five times before termination on the 20th day. As expected, the SFN@RB@SPM-treated group manifested the weakest fluorescence emitted from the tumor tissue among all the groups (Figure 5B). The parameter for tumor quantification also indicated that the mice in the SFN@RB@SPM-treated group displayed the smallest tumor area (Figure 5C) and avoided a significant loss of body weight (Figure 5D). We also conducted H&E staining for the tumor slice of each group and, surprisingly, found that only in the SFN@RB@SPM-treated group, was there a robust infiltration of neutrophils adjacent to the tumor tissue (Figure 5E). This phenomenon suggests that the synergistic anti-glioma effect of SFN@RB@SPM with SDT might be due to the coordination of SFN-induced GSH depletion and SDT-induced high activation of neutrophils.

## 4. Discussion

Organic sonosensitizers have an easier synthesis procedure, excellent biodegradability and higher yield of ROS than the inorganic ones [14]. Even though Rose Bengal (RB), an organic sonosensitizer that belongs to Xanthene, has been widely applied in photodynamic therapy (PDT) due to its strong photostability [14], studies have demonstrated that RB could cause more apoptosis of tumor cells when sensitized by US rather than by light [14]. Apart from the difference in cytotoxicity, US also shows a deeper penetrability than light [42,43]. Also, US-targeted microbubble destruction can instantly disrupt the BBB to increase the drug concentration in situ [44].

Although SDT is a promising approach for treating deep-seated tumors, it is still urgent to modify the existing sonosensitizers or to establish a combination therapy with other antitumor chemicals to improve SDT efficacy. The key point is to find a suitable carrier for delivering antitumor drugs used in SDT. Recently, liposomes, which also morphologically resemble the spherical nanomicelles used in this study, are regarded as the fourth-generation drug delivery system for SDT due to their high biocompatibility, sufficient drug-loading capacity, stable structures for hindering a drug’s degradation, and easy procedures for modification with tumor-specific probes [45,46]. However, the liposome-induced cytotoxicity to normal cells is inevitable [47,48,49]. To reduce such side-effects of lipo-carriers, as well as to gain more advanced features like low immunogenic reactivity, amphiphilic capability, and tumor-homing ability for enhancing drug delivery [25,26,27,28,50], we fabricated a novel nanocarrier for SDT called a self-assembled nanomicelle. The peptide of C_18_GR_7_RGDS [41,51], with the introduction of hydrophilic (RGDS) and hydrophobic (C_18_) terminals on the spacer ends of R_8_, tends to form a spherical-like supramolecule by noncovalent interactions [52,53]. This peptide amphiphile can perform α_v_β_3_-overexperssed glioma-targeting [24,54], and has also been applied in treating various malignancies [51] due to its enhanced drug-loading capacity, prolonged retention in circulation, and increased accumulation in tumor tissues [41]. Thus, in this study, we chose the amphiphilic peptide C_18_GR_7_RGDS (abbreviated as SPM) as the nanocarrier for drug delivery.

Sulforaphane (SFN), one of the most widely investigated isothiocyanates that is abundant in cruciferous vegetables [55], has been found to be a very potent chemopreventive agent for its effect of inhibiting procarcinogen metabolism, induction of apoptosis and inhibition of cell cycle progression [56,57,58,59,60,61,62] in numerous cancers [63]. Among many mechanisms by which SFN promotes apoptosis in cancer cells, facilitating ROS production is one of the most relevant [34,35,39,64,65,66]. Accumulating evidence from our study and others has demonstrated that SFN resembles a hermetic agent [67], a compound that is able to induce completely opposite effects depending on its concentration or the cell types [55,68,69,70,71,72]. Namely, SFN is chemopreventive and antioxidative at low doses, while it is cytotoxic to tumor cells at higher doses [68]. SFN can also permeate the BBB and exert an ROS-dependent anti-glioma activity [35,73,74].

**Figure 6:**
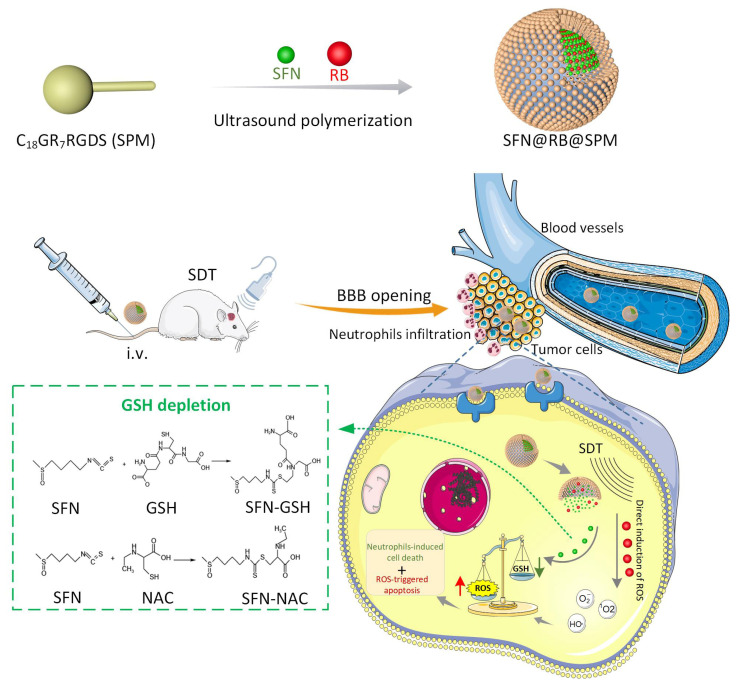
A diagram illustrating the potential mechanism by which SFN@RB@SPM synergistically improves the efficacy of traditional SDT. SFN and RB are encapsulated into a self-assembled nanomicelle (C_18_GR_7_RGDS) under ultrasound polymerization. The orthotopic glioma-bearing nude mice were intravenously injected with SFN@RB@SPM, and after 2 h, the mice were treated with SDT. The RGDS-motif guides the nanocomplex to cross the BBB and further penetrate the tumor tissues through SDT-induced transient BBB-opening. While approaching the tumor cells that express the specific receptor for the RGDS-motif, SFN@RB@SPM is internalized and releases the payload comprising SFN and RB. RB may directly promote the generation of ROS by an SDT-induced cavitation effect like the pyrolysis of water within the tumor cells. In addition, the released SFN can react with GSH or its precursor NAC to form SFN-GSH or SFN-NAC, both of which can significantly diminish the GSH level. Furthermore, the instant BBB opening and the massive numbers of ROS may activate neutrophils, which further execute the antitumor function and synergistically augment the SDT effect.

The efficacy of SDT is determined by the mechanical effect of US irradiation and the chemical effect of tumor-killing ROS. RB is a regular sonosensitizer that can induce ROS under US irradiation. To further elevate the generation of ROS and limit the scope of induced ROS to the tumor site as precisely as possible, we considered SFN as an ideal candidate for its high potential for inducing ROS through the GSH-depletion mechanism (Figure 6), and SPM were used to enhance tumor targeting efficiency. Therefore, to maximize the SDT efficacy, in this study, we wrapped it into SPM together with RB to construct a novel nanocomplex named SFN@RB@SPM. As shown in Figure 1, these novel nanocomplexes are homogeneously spherical in PBS solution, with an average diameter of ~57 nm and remain stable within 15 days. Moreover, in vitro experiments showed that the SFN@RB@SPM-treated group displayed a high rate of cell uptake (Figure 2), and the highest cytotoxicity (Figure 3) under US irradiation. Even though SFN can trigger cell apoptosis through elevating intracellular ROS levels [39,64,75], in our study, at the dose of 10 μM, SFN alone was insufficient to cause massive cell death (Figure 3A,C) and the ROS level was too low to detect (Figure 4A). These results were consistent with other studies which found that SFN usually shows a robust cytotoxicity above the dose of 10 μM [35,36,37,38,40,64]. However, compared with the other groups, the nanocomplex SFN@RB@SPM significantly enhanced the cytotoxic effect (Figure 3A,C) under US irradiation, suggesting that SFN can boost the SDT efficacy in vitro though synergistically triggering apoptosis with RB. Intracellular glutathione (GSH) is the key molecule to counteract excessive ROS within the tumor cells. As previously reported by others, SFN can conjugate with GSH to form SFN-GSH or SFN-N-acetylcysteine (SFN-NAC) through the mercapturic acid pathway [74,76,77]. The formation of SFN-GSH and SFN-NAC may competitively inhibit the antioxidant function of GSH and NAC. Thus, we corroborated the findings of other studies that SFN induces tumor cell apoptosis through depletion of GSH and subsequently excessive ROS generation [35,78,79,80]. In our study, the SFN- and RB@SPM-treated groups could only induce small amounts of ROS, which seldom showed influence on the cell viability under the condition of non-US irradiation. However, under US irradiation, both the RB@SPM and SFN@RB@SPM treatment groups showed increases in intracellular ROS. The SFN@RB@SPM-treated group had a more significant increase, and the cell viability in this group was the lowest. It seems that the cell viability was inversely proportional to the intracellular ROS level. In our study, neither SFN nor RB@SPM alone could induce sufficient ROS to inhibit cell viability under non-US irradiation conditions. However, when SFN was encapsulated into the nanomicelles (SFN@RB@SPM), the ROS level was slightly increased, and the cell viability was decreased accordingly (Figure 4A,C). Under non-US conditions, compared with SFN-treated group, the gentle elevation of ROS level in the SFN@RB@SPM-treated group might be due to a higher efficiency of SFN delivery to the cells mediated by the nanomicelles. Consistent with previous studies [35,78,79,80], we also confirmed that SFN can inhibit the intracellular level of GSH because such an GSH-dependent decrease in cell viability can be reversed by pretreating cells with NAC (Figure 4B,C). However, the GSH level in the RB@SPM-treated group had no significant change under US irradiation (Figure 4B), while the decreased cell viability of the SFN@RB@SPM-treated group could only be partially restored by pretreating with NAC (Figure 4C). Hence, we speculate that the increased generation of ROS may not fully be attributed to the depletion of GSH, but results partially from ultrasonic cavitation (e.g., pyrolysis of water caused by collapse of cavitation bubbles) [81]. Although SFN and RB exhibited different mechanisms to facilitate the generation of intracellular ROS, their combined effect enhanced the SDT efficacy, which was demonstrated by the observation that the SFN@RB@SPM treatment resulted in the lowest cell viability under US irradiation (Figure 4C).

To further verify the synergistic effect of SFN and classical RB-mediated SDT in vivo, we applied SFN@RB@SPM to glioma-bearing nude mice under SDT. As expected, SFN@RB@SPM displayed the most inhibition of tumor growth. Pathological staining of the brain slices also showed the highest rate of tumor cell death in the SFN@RB@SPM-treated group. The most interesting phenomenon was that only in the SFN@RB@SPM-treated group, a huge number of neutrophils infiltrated into the perivascular space around the tumor site (Figure 5). It has been reported that immune cells rarely appear in the brain parenchyma due to the existence of the blood–brain barrier (BBB) and blood–cerebrospinal fluid barrier (BCSFB) that hinder the entry of immune cells to the brain [82,83]. It is widely accepted that the innate immune system acts more promptly than the adaptive immune system when the body encounters detrimental stimuli like infection and cancer.

At the early stages of glioma, the tumor microenvironment (TME) consists of a variety of non-tumor cells, including innate immune cells, stromal cells and vascular cells [84,85], while myeloid cells constitute the majority of these cells [86]. Considering the innate immune system, most previous studies have focused more on tumor-associated macrophages (TAMs), whereas insufficient attention has been paid to the tumor-associated neutrophils (TANs), despite their preponderance in circulation [86]. Early studies suggested that tumor-associated neutrophils (TANs) were just bystanders with little function, namely, either circulating transiently through the tumor vasculature or passively accumulating in the stroma [86]. However, emerging evidence indicates that TANs play an active role in interacting with the TME [87,88]. Particularly, with the deepening of glioma study, neutrophils have been found to play a crucial role in glioma progression [89], although whether their role involves immune-suppression or immune-enhancement remains controversial [86,90]. Although some studies show that the peripheral ratio of neutrophils-to-lymphocytes has diagnostic and prognostic value [89], the exact local function of neutrophils at the tumor site remains unclear. In our study, the phenomenon that massive numbers of neutrophils appeared in the tumor site can be explained by the fact that neutrophils are chemoattracted by large amounts of ROS released in the tumor site, which are triggered by SFN@RB@SPM under SDT. The neutrophils then eliminate tumor cells by directly contacting them and producing more ROS in a positive-feedback process called Respiratory Burst [91,92,93,94]. Furthermore, it may be anticipated that with the progression of the glioma, more immune cell populations would be recruited to the primary tumor site or the distant tumor site of metastasis, under which situation the neutrophils may function as the antigen-presenting cells to mediate downstream adaptive immune cell-mediated cytotoxicity [86,95]. Because the nude mice used in our study are deficient in T-cells development, the innate immune cells accordingly play a central role in eliminating tumor cells. Therefore, we can conclude that the neutrophils might be recruited into the tumor site to execute their antitumor function via instant BBB-opening, and the massive ROS levels generated through the combinatory induction of RB and SFN during SDT may also facilitate the migration and accumulation of neutrophils to enhance their antitumor effect.

## 5. Conclusions

In summary, we upgrade the traditional sonosensitizer used in SDT by encapsulation of the antitumor compound SFN and regular sonosensitizer RB into the self-assembled nanomicelle SPM. By this modification, we achieved two goals: (1) enriching the local concentration of SFN within the tumor cells by targeted delivery of SPM; and (2) utilizing complementary mechanisms (GSH depletion and innate immunity reactivation) to synergistically enhance SDT’s tumor-killing efficacy.

## Data Availability

Data will be made available on request.

## Supplementary Materials

The following supporting information can be downloaded at: https://www.mdpi.com/article/10.3390/pharmaceutics17010034/s1. Figure S1: Characterization of RB@SPM; Figure S2: Tumor-targeting efficiency of SFN@RB@SPM. Figure S3: In vivo image reflecting drug enrichment at tumor site in glioma-bearing mice.
