# Supplementary material for: Sulforaphane Wrapped in Self-Assembled Nanomicelle Enhances the Effect of Sonodynamic Therapy on Glioma
Source: Pharmaceutics. 2024 Dec 30;17(1):34. doi: 10.3390/pharmaceutics17010034 (PMC11769538; doi:10.3390/pharmaceutics17010034)
Supplement: Supplementary file 1 [file pharmaceutics-17-00034-s001.zip › pharmaceutics-3351245-supplementary.pdf]

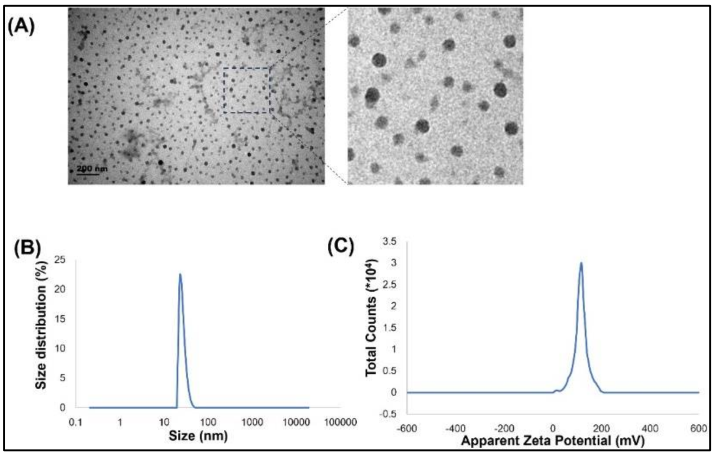

Figure S1. Characterization of RB@SPM.

(A) The nanocomplex RB@SPM was visualized by transmission electron microscope with 80000x magnification. Scale bar, 200 nm. (B) Size distribution indicates the mean size of RB@SPM is ~50 nm. (C) the apparent zeta potential of RB@SPM is 132±0.74 mV.

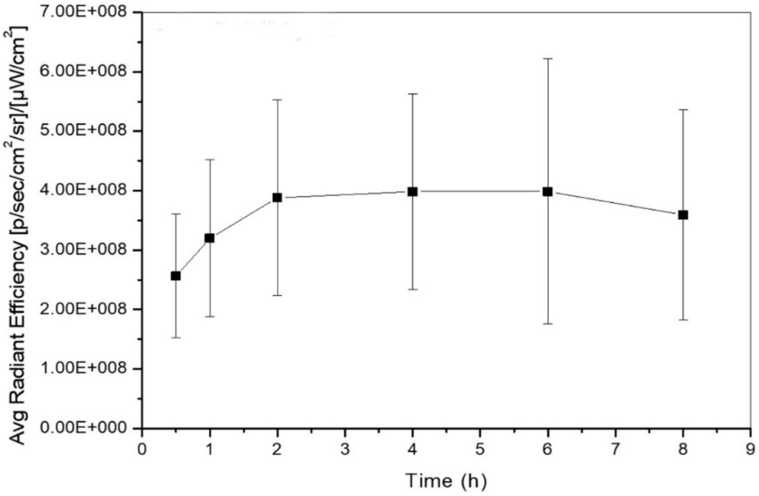

Figure S2. Tumor-targeting efficiency of SFN@RB@SPM.

The U87-MG cells-derived glioma-bearing mice were intravenously injected with SFN@RB@SFN (normalized to the dose of 1.77mg/kg of SFN). At the time points of 0.5 hour, 1 hour, 2 hours, 4 hours, 6 hours, and 8 hours, the fluorescence that reflects the concentration of SFN within the tumor cells were recorded by IVIS Spectrum imaging system.

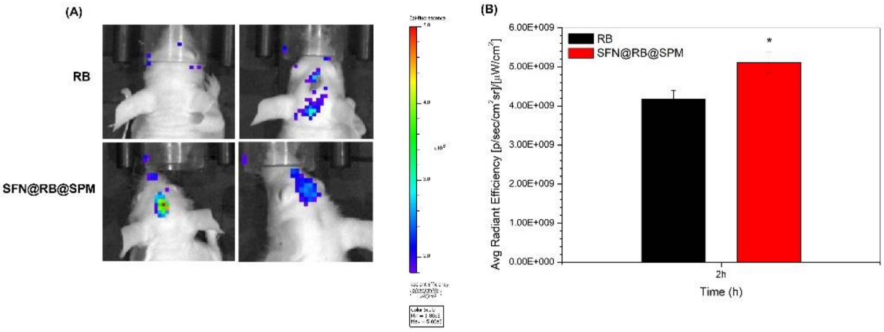

Figure S3. In vivo image reflecting drug enrichment at tumor site in glioma-bearing mice.

(A) Two hours after i.v. injection, the delivery of free RB and SFN@RB@SPM to the xenografted glioma were indicated by the intensity of the fluorescence of RB. (B) Quantification of the fluorescence intensity in (A).
